# MAiD for geriatric syndromes: Special considerations

**DOI:** 10.1177/08404704221141048

**Published:** 2023-02-20

**Authors:** Caitlin Lees, Melissa K. Andrew

**Affiliations:** 1 12361 Dalhousie University, Halifax, Nova Scotia, Canada.

## Abstract

Medical Assistance in Dying (MAiD) brings unique considerations in the context of geriatric syndromes such as frailty and cognitive or functional impairment. These conditions are associated with complex vulnerability across health and social domains and often do not have predicable trajectories or responses to healthcare interventions. In this article, we focus on four categories of gaps in care that are particularly relevant for MAiD in geriatric syndromes, namely, inadequacies in access to medical care, appropriate advance care planning, social supports, and funding for supportive care. We conclude by arguing that appropriately situating MAiD in the context of care for older adults requires careful consideration of these gaps in care to enable real, robust, and respectful healthcare choices for people living with geriatric syndromes and approaching end-of-life.

## Introduction

Since 2016, the legalization of Medical Assistance in Dying (MAiD) in Canada has meaningfully changed how Canadians approach end-of-life and aging. For individuals who feel their quality of life is so poor that they no longer wish to continue living, a medically assisted death is now an option, provided they meet specific legal criteria.^1,2^ Initially, the criteria allowed access to MAiD in cases where the individual was aged 18 or older, had decision-making capacity, was eligible for publicly funded healthcare services, was making a voluntary request without coercion or external pressure, provided informed consent to receive MAiD, was in an “advanced state of irreversible decline in capability,” and experiencing “enduring and intolerable” suffering not alleviated under conditions acceptable to the patient, and where there was a “grievous and irremediable” condition where natural death was felt to be reasonably foreseeable.^1^ The criteria were then amended in March 2021 to broaden access to those in whom death is not felt to be reasonably foreseeable. The amendment also allows for a waiver of final consent for the procedure in the case of loss of capacity, such that a person, once deemed eligible and if natural death is felt to be reasonably foreseeable, may make an agreement with a MAiD practitioner to undergo the procedure on a certain date even if capacity has been lost.^2^

Medical assistance in dying is an important issue for older adults; Canadian experience shows that 83.3% of MAiD deaths in 2021 occurred in people aged 65 and older.^3^ Some reasons for this are likely to be self-evident (e.g. the fact that most health conditions underlying MAiD requests increase in prevalence with age), while others may be less obvious (e.g. evolving views on quality of life, value placed on independence and not being a burden, challenges accessing health and social care, and social isolation as peers become ill or die).^3,4^ As access to MAiD expands, and given how many older adults experience a medically assisted death, it is important to examine more fully how best to ensure that MAiD can be appropriately included in a suite of health and social care options for older adults. This is of particular relevance given that these same individuals may live with vulnerability and often experience disproportionate difficulty accessing other needed care or supports to ensure basic quality of life. Those with geriatric syndromes are particularly vulnerable to gaps in care, including inadequate access to medical care, appropriate advance care planning, social supports, and funding for supportive care.^5^ Indeed, challenges accessing MAiD itself could be included in this list of gaps in care for some populations and groups. Geriatric syndromes are age-associated and thus they are most often, though not always, experienced in older age. The term older adult is used here in a way that is meant to be inclusive of people who are both *older* (often defined as age 65 and older), while allowing that some people living with geriatric syndromes are under age 65. Geriatric syndromes include complex and multi-factorial conditions such as frailty, dementia and cognitive impairment, and impairments in physical or sensory function; importantly, these conditions are often associated with impacts on quality of life, social vulnerability, and/or isolation that may also influence the desire for MAiD.^6,7^ The expansion of MAiD creates some urgency in addressing these gaps in care, such that those who opt for MAiD are making a choice to die due to intolerable suffering despite adequate access to alternative means of management and palliation, rather than having no other option to end suffering. In this article, we discuss each of these gaps in turn and conclude that addressing these gaps must go hand-in-hand with ensuring robust access to MAiD.

## Access to care

For people living with geriatric syndromes, access to care may be a challenge due to lack of diagnosis, or once diagnosed, lack of available services, or lack of timely, appropriate referral to such services. Even when services are available, accessibility can be further affected for those with sensory, mobility, functional, and/or cognitive impairments which may make arranging and coordinating appointments and getting out of one’s home, including securing transportation, a challenge. As Canadians continue to struggle with access to primary care,^8^ recognition of a geriatric syndrome without longitudinal care through a primary care provider is not without challenges.^9^ In the case of frailty, the incremental effect that it has on survival and adverse outcomes including disability and institutionalization makes its recognition and diagnosis important as it allows for the recognition of care needs and provision of advance care planning.^10^ In the case of dementia, under-diagnosis is recognized as an important barrier to accessing community supports and medical care that support quality of life.^11‐15^

Once diagnosed, those with geriatric syndromes often do not have a clear trajectory for either symptom progression or care. In contrast, individuals diagnosed with advanced malignancy tend to have more predictable trajectories in health and function and are generally well served, where available, by clear care pathways with increasingly standardized care across Canada, coordinated through cancer centres.^16^ The implementation of standardized, evidence-based approaches for management of dementia and frailty will be key to ensure equitable access to high quality care for Canadians with these conditions, in line with recommendations of *A Dementia Strategy for Canada* and as recommended by the Canadian Frailty Network.^17,18^

It is an important balance to strike in appropriately resourcing MAiD, while also ensuring access to the health and social care for those with geriatric syndromes. In order to ensure that end-of-life choices are real, robust, and respected, it should not be the case that one pathway to end-of-life care, either natural or medically assisted, is easier to access than the other.

## Advance care planning

Advance Care Planning (ACP) can be done either proactively before the diagnosis of any specific condition or after a diagnosis is made. Once a diagnosis is made, ACP is ideally done early in the trajectory of the disease or condition, with some evidence showing that advance care planning can improve the quality of end-of-life care,^19^ though evidence for how ACP affects quality of life is limited. For people living with chronic disease, including a diagnosis of a geriatric syndrome, we argue that early ACP is key, such that people and their loved ones are given a chance to reflect upon what makes a life worth living for them, and an opportunity to feel empowered and engaged in making decisions about whether healthcare interventions designed to extend life are in keeping with their values. For example, a frail elderly person with recurrent infections and poor quality of life may opt to decline antibiotics in the future. Ongoing medicalization and the unquestioning treatment of medical issues in people living with comorbidities may contribute to a creep of lower quality of life; this in turn may affect wishes about MAiD. Older people deserve a clear understanding of their prognosis and likely disease trajectory, and to understand that they can opt to stop prolonging life at any time, rather than treatments and interventions aimed at prolonging life (but often not succeeding in promoting quality in that life) simply being a default. Such decisions may improve individual autonomy around end-of-life decisions, with options that could include natural death related to an acute medical event that is palliated rather than being aggressively treated, ongoing life-prolonging treatment with a natural death, or MAiD.

## Social supports

Social vulnerability or isolation and geriatric syndromes often travel together. Dementia offers a useful example. One area of focus of the dementia strategy for Canada is to ensure Canadians are able to access holistic care and programs, including “social, behavioural, and psychological supports,” as well as dementia-inclusive communities.^17^ Similarly, community care capacity and holistic care across a range of settings are listed as part of the Framework on Palliative Care in Canada.^20^ Access to holistic care, including a supportive community and social supports, is key to ensuring that people living with geriatric syndromes can access appropriate health and social care to optimize quality of life. Without broad, equitable access to such supports, people requesting MAiD with a geriatric syndrome as their underlying condition may (or may not) be aware of alternative means to alleviate their suffering, but these may not be meaningfully available to them.

## Adequate funding of supports for symptom management

During the initial period of legal access to MAiD in the case of a reasonably foreseeable natural death, concern was expressed about the potential for people to undergo MAiD due to lack of access to quality palliative care that might otherwise alleviate suffering. Fortunately, this does not appear to have materialized in a substantial way, with most recent statistics showing that the majority (80.7%) of MAiD recipients received palliative care, and 88.0% had access to palliative care if needed. Further, it is unclear as to whether these statistics capture access to the high quality primary palliative care that many practitioners other than formal organized palliative care services can provide.^3^ As access to MAiD has since expanded to include those in whom death is not reasonably foreseeable, similar concerns have arisen about whether MAiD will be sought when other options have been left unexplored or found to be unavailable.^21^ While Canada does have publicly funded healthcare, not all services in support of wellness are publicly funded and/or even available in all jurisdictions across the country—and this is particularly so for people living with geriatric syndromes.

Comparisons between conditions can be illuminating. In general, the expected symptoms for a person living with advanced malignancy or organ failure are seen as “medical” (e.g. shortness of breath, pain, and nausea) and are reasonably well covered by publicly funded health services. Meanwhile, the expected symptoms for a person living with dementia (e.g. memory impairment, need for assistance in activities of daily living) are seen as “social” and are often not covered by health services. Managing these symptoms becomes the responsibility of the person and family and comes with costs in informal (unpaid, often friends and family) caregiver time and potential caregiver burden and/or formal (paid) supports either provided in home or institutional settings.^22,23^

Given that home care and nursing home care are not covered by the *Canada Health Act*, depending on policies in place in individual provinces, “public” nursing home care costs upwards of $3,000/month.^24^ Care in the home, both in terms of Home Support Worker time and any safety devices that are required, can also be costly. Although in most areas of Canada these costs can be mitigated by income-based subsidies, the cost to families remains substantial and may be prohibitive. Consider for example a relatively common case arising in couples who are married, living common-law, or in a domestic partnership in which one spouse requires nursing home admission while the other spouse wants to stay in the family home. If the spouse going to nursing home is the main income earner whose income is used for the means testing, a substantial percentage of the household income might need to go towards the cost of nursing home care, leaving the spouse at home without enough income to make ends meet. Clearly, there are also important gender issues at play, with women more often providing informal care and women in older cohorts being less likely to have had opportunities to work outside the home in order to have income independent of their spouse.^25–27^ These issues are also applicable to older 2SLGBQIA + individuals, who may have added challenges of living with stigma and marginalization past and present and potentially having relationships that are not recognized legally or socially in the wider community. This remains an important area for further study.

Clearly, this required care may be difficult for many older people and their families to afford, and as a result, they may be unable to access the supports necessary to optimize quality of life in the setting of geriatric syndromes. Indeed, financial pressures, and the worry that the high costs of one’s care for a geriatric syndrome will leave family members disadvantaged or even impoverished, may contribute to concerns about voluntariness and coercion in decision-making around MAiD. In this sense, the expansion of MAiD under Bill C-7 creates a pressing need to ensure that there is adequate treatment for both physical symptoms and social concerns created by geriatric syndromes, such that MAiD truly is accessed only when a person experiences intolerable suffering despite adequate and appropriate access to alternative means to alleviate suffering.

## Conclusion

The possibility of access to MAiD has been welcomed by many older people including those living with geriatric syndromes. Even so, it remains important to situate MAiD in the suite of options for people living with geriatric syndromes. As we have discussed, geriatric syndromes including frailty and dementia bring complex health and social care needs which are unfortunately subject to gaps in access to care, advance care planning, social supports, and funding for symptom management. As MAiD is now part of the healthcare landscape, it is essential to ensure that people living with geriatric syndromes in Canada can fully exercise their autonomy in decision-making. This necessitates that appropriate access to MAiD is maintained, while policy-makers and leaders ensure these gaps in care are addressed such that health, social care, and quality of life are optimized for older adults in Canada.
